# A Review of Complementary and Alternative Treatments for Autism Spectrum Disorders

**DOI:** 10.1155/2012/870391

**Published:** 2012-11-28

**Authors:** Nicholas Lofthouse, Robert Hendren, Elizabeth Hurt, L. Eugene Arnold, Eric Butter

**Affiliations:** ^1^Department of Psychiatry, The Ohio State University, 1670 Upham Drive, Columbus, OH 43210, USA; ^2^Department of Psychiatry, University of California, San Francisco, CA 94143-0984, USA; ^3^Nisonger Center, The Ohio State University, 1670 Upham Drive, Columbus, OH 43210, USA; ^4^Nationwide Children's Hospital, Columbus, OH 43081, USA

## Abstract

Given the severe and chronic problems associated with Autism Spectrum Disorders (ASD) and the limitations of available treatments, there exists a large public health need for additional interventions. As more parents are inquiring about complementary and alternative treatments (CATs), both parents and practitioners require up-to-date information about them and whether and how to integrate them into treatment. After presenting data on CAT usage patterns for ASD, we review 13 ingestible (i.e., orally administered) and 6 noningestible (i.e., externally administered) CATs for ASD. For each CAT we briefly describe its definition; rationale for use; current research support, limitations, and future directions; safety issues; and whether we currently recommend, not recommend, or find it acceptable for the treatment of ASD. We conclude this paper with recommendations for future research and ten clinical recommendations for practitioners.

## 1. Background and Significance

Many treatments (Txs) have been proposed for Autism Spectrum Disorders (ASD) with the most effective being combined Tx involving specialized and supportive educational programming, communication training (e.g., speech/language therapy), social skills support, and behavioral intervention [1, 2]. Occupational and physical therapy also may promote progress by addressing comorbid difficulties of motor coordination and sensory deficits [3]. Behavior modification (e.g., applied behavior analysis [ABA]) has the most empirical support for a single Tx, with documented improvements in language, social, play, and academic skills, and reduction in severe behavioral problems [4]. However, behavioral Txs are time and staff intensive, requiring up to 30–40 hours of Tx per week for several years by trained staff working directly with the child and typically focusing on one or a few behaviors at a time. 

 Risperidone (Risperdal) and aripiprazole (Abilify) are the only FDA-approved medications for ASD, and they are approved only for the Tx of irritability in 5–16 year olds with ASD. No medications are currently established to treat ASD core symptoms. “Off-label” medications are often prescribed for cooccurring behaviors such as inattention, impulsivity/hyperactivity, sleep problems, repetitive/perseverative behaviors, anxiety, mood, agitation, aggression, and disruptive and self-injurious behaviors but may have significant side effects [5]. Survey research has estimated the utilization of psychotropic medication for youth with ASD as high as 47% [6], but there is ongoing debate about the role of such agents [7]. Response rates to medication for comorbid diagnoses in children with ASD may be lower than for children without ASD; for example, the response rate of methylphenidate for typically developing children with Attention-Deficit/Hyperactivity Disorder (ADHD) is 70% [8] but for children with ASD and ADHD symptoms it is only 50% [9]. Complementary and alternative medicines are also commonly reported, but their effectiveness remains unproven [10]. Therefore, given the limitations of available Txs for ASD (expense, effort, risk, less than perfect response) and the severe and chronic nature of ASD, there is a large public health need for additional interventions.

The National Center for Complementary and Alternative Medicine (NCCAM) defines complementary and alternative medicine (CAM) as “a group of diverse medical and health care systems, practices, and products that are not generally considered to be part of conventional medicine” [11]. Because these interventions can include both ingestible (i.e., orally administered) and noningestible (i.e., externally administered) Txs we refer to them collectively as complementary and alternative Txs (CATs). They are complementary when these practices are “used together with conventional medicine,” and alternative when “used in place of conventional medicine.” However, to be a truly designated complimentary Tx, incremental effects when added to conventional Tx should be empirically demonstrated. Likewise, to be truly designated an alternative Tx, similar effects when compared to conventional Tx should be demonstrated. Few CATs for any psychiatric condition (and none for ASD) fulfill these requirements, so the majority are not valid CATs but merely “wanna-be” CATs!

Global studies report rates of CAT use for ASD range from 32 to 87% in the US [12–15], 52% in Canada [16] and 41% in China [17]. Even before receiving a diagnosis of ASD for their children approximately one-third of parents were already using dietary CATs [14]. 

In the US, most parents report concerns regarding medication safety (84%) and side effects (83%) as the main reasons for choosing CATs [12]. CATs are perceived as a risk-free approach that may improve a child's outcome [18]. Initial referral sources in the US tend to be a physician or nurse, 44%, with “other parents” next at 16% [13]. In Canada, these include friends/family 35%, occupational therapists 27%, physicians 23%, Internet 23%, or books 15% [16]; in Turkey, other parents 30%, Internet/books 24%, scientific journals 22%, or physicians 20% [19].

 Regarding the number of CATs used in 2008, the Interactive Autism Network's (IAN) ongoing online survey of 1000's of U.S. parents reported a total of 381 different Txs, most of which are CATs, being used at any one time, with an average of five Txs per child (min. = 0, max. = 56 concurrent Txs!), >50% receiving ≤4 Txs, and 5% receiving no Tx at all [20]. 

 In terms of which specific CATs are used, applying NCCAM's five categories, Hanson et al. [12] reported the following percentages for a US sample: Biologically-Based Therapies (e.g., herbs, foods, and vitamins) 54%, Mind-Body Interventions (e.g., meditation) 30%, Manipulative or Body-Based Methods (e.g., massage) 25%, Energy Therapies (e.g., Reiki or electromagnetic fields) 8%, and Alternative Medical Systems (e.g., homeopathy) 1%. However, usage in other countries/cultures may be different as shown by Şenel et al. [19] in Turkey (vitamins and minerals 84%, special diet 79%, sensory integration 77%, other dietary supplements 50%, and chelation 50%) and by Wong [17] in China (acupuncture 47.5%, sensory integration 42.5%, and Chinese Medicine 30%).

There is limited scientific evidence of efficacy for some CATs, but research on CATs for ASD is imperative because key safety and efficacy questions remain for the majority [11]. Berman and Straus [21] observed that many CAT studies assume that Txs are well defined, including optimal dose/duration/intensity, that the sample has been correctly diagnosed and selected, and that the Tx is consistent from one practitioner to another. They note that CATs should meet the same fundamental requirements as for conventional Txs, using the same tools and techniques as those for conventional research to isolate the specific effects from the nonspecific effects of Tx as much as possible. Such controls include rigorous protocols, randomized controlled trials (RCTs) with, where possible, placebo/sham control conditions with double-blind designs, and careful diagnosis. Such research is vital because, even though people often assume CATs, particularly natural ones, are safe, their use without supportive evidence is risky because they may have dangerous, sometimes life-threatening and irreversible side-effects; fail to reduce symptoms or improve functioning in patients with severely impairing disorders; delay use of other more established Txs; and/or waste families time, energy, and money.

As of December 2011, there have been 14 comprehensive reviews of ASD CATs in Medline and PsychInfo [3, 18, 22–32], and 5 Cochrane reviews of specific ASD CATs (acupuncture [33]; music therapy [34]; omega-3 [35]; gluten/casein-free diets [36]; and B6-magnesium [37]). 

The CATs summarized in this paper (and in Tables 1–3) do not exhaust all those that have been tried or advocated for ASD, but are those for which some positive research evidence exists (Table 4 lists CATs either without positive effects in RCTs or without sufficient evidence to include in this paper). We have not included off-label drugs, which could be technically classified as an alternative Tx, because they are usually considered as “conventional” Txs rather than CATs. The included CATs were identified via Medline and PsychInfo title searches of key terms up to December 2011. For practical purposes we have organized CATs under two main sections: A. Ingestible (*N* = 13) and B. Noningestible (*N* = 6). For each CAT we briefly describe its definition; rationale for use; current research support, limitations, and future directions; safety issues; and expected clinical treatment outcomes, with the caveat that such expectations are more likely to occur if the CAT is administered in the same manner as in the research study and if the patient has similar characteristics to the research sample. To help clinicians to decide whether to use a CAT or not, we also apply a clinical guideline: Txs that are Safe, Easy, Cheap, and Sensible (SECS) do not require as much evidence to justify an individual patient trial as do Txs that are Risky, Unrealistic, Difficult, or Expensive (RUDE) [38]. Currently, none of these CATs have enough empirical support to be considered a stand-alone Tx for ASD. See Table 1 for a summary of commonly used ingestible and noningestible CATS, Table 2 for a summary of RCTs on ingestible CATS and Table 3 for a summary of RCTs on noningestible CATS. 

## 2. Ingestible CATs for ASD 

### 2.1. Melatonin

 Melatonin is an endogenous neurohormone released by the pineal gland in response to decreasing levels of light, it causes drowsiness, and sets the body's sleep clock. ASD has a high frequency of sleep problems and melatonin is increasingly used to help children with ASD fall asleep [39, 40]. Rossignol and Frye [41] published a review and meta-analysis of 35 studies and reported that 9 studies of melatonin levels reported at least one sleep abnormality (7 low, 2 high, 4 circadian); 4 studies reported significant correlations between melatonin levels and ASD symptoms; and 5 studies reported gene abnormalities associated with decreased melatonin production. Of 18 Tx studies, 13 were uncontrolled, 5 were randomized, double-blind, placebo-controlled crossover trials, and 6 studies of night-time administration led to improvements in daytime behavior. Within these 5 RCTs (*N* = 61, 2–18 yrs old, 2–10 mg/day), melatonin was associated with increases in sleep duration (44 min, ES = 0.93) and decreases in sleep onset latency (39 min, ES = 1.28), but nighttime awakenings were unchanged. Side effects were minimal to none. 

 Unfortunately, small sample sizes, variability in sleep assessments, and lack of follow-up limit the conclusiveness of these studies but, overall, melatonin is one of the best studied CATs for ASD. Future research directions include using placebo controlled or comparative effectiveness trials to determine which sleep intervention works best for which child, larger samples identifying inexpensive Tx targets to better match melatonin and other Txs to the individual with ASD, combining melatonin with other Txs for insomnia and ASD, and, based on one of the author's clinical experiences, identifying those with mid and late insomnia who might respond to higher doses of melatonin. Melatonin is sensible, easy, cheap, and safe; therefore, we recommend a trial of melatonin for sleep delay problems in ASD. 

### 2.2. B6 and Magnesium

 One of the oldest and best studied dietary supplementation strategies for ASD is high-dose pyridoxine (vitamin B6) and magnesium (Mg), presumably correcting a metabolic aberration that requires higher than usual intake of those essential nutrients. Improvements maybe noted in social interactions, communication, and stereotyped, repetitive behaviors although the measurements for these symptoms are impressionistic. Pfeiffer et al. [42] identified a dozen studies demonstrating improvements, most controlled in some way, but with many methodological flaws. Reports date back at least to Rimland's 1973 anecdotal summary [43] suggesting clinically significant benefit. By 1978 a double-blind placebo-controlled withdrawal study of apparent responders [44] reported greater disturbance in autism symptoms upon placebo-masked withdrawal of B6 than upon continuing it, but this could have resulted from an induced B6 dependency [45]. 

 In a prospective open trial [45], 15 of 44 children aged 3–16 with severe ASD (34 on psychotropic medication) responded to B6 30 mg/kg/day (600–1,125 mg/day) +Mg lactate 400–500 mg/day with increased alertness and reduction of outbursts, negativism, self-mutilation, and stereotyped behavior. All but one deteriorated on withdrawal. The prototypical responder was a young small-for-age male. Thirteen responders and 8 nonresponders entered a double-blind crossover with placebo (2 weeks each, random order), and 10 responders versus 2 nonresponders showed more improvement on B6/Mg than on placebo (*P* < .01). 

 However, a double-blind placebo-controlled study reported no benefit in 10 children with autism treated for 10 weeks [46]. A study of 60-day hospital patients with ASD aged 3–14 involved 4 crossover trials with each trial lasting 8 weeks: 2 wk baseline, 2 wk first Tx, 2 week 2d baseline, 2 wk 2d Tx) [47]. Doses were B6 30 mg/kg/day up to 1 g/day, and Mg 10–15 mg/kg/day. The first crossover (*N* = 16) showed improvement for both the combination and Mg alone. In the second (*N* = 21), comparison of the combination versus placebo, they reported significant improvement for the combination, but the placebo comparison was not shown. In the 3rd (B6 versus placebo, *N* = 35) and 4th (Mg versus placebo, *N* = 37), there was negligible difference between the active and placebo conditions. In an open study (*N* = 33), significant improvements in ASD symptoms were reported from B6 0.6 mg/kg/day and Mg 6 mg/kg/day for 6 months, with return of symptoms within a few weeks of discontinuation [48]. Improvement was associated with increase towards normal erythrocyte Mg.

 In sum, the evidence for B6 + Mg from over 25 studies remains rather equivocal, a bit more positive than negative. Despite encouraging results from open studies, those from RCTs are less promising. Limitations of extant research to be addressed by future research include small samples, inconsistent diagnostic methods and assessments, lack of evidence for mechanism, and lack of blinding. Future studies should involve larger, double-blind placebo-controlled trials using a biomarker of Tx response such as B6 and Mg levels. It is probably safe if the daily doses of B6 are kept well below a gram and daily doses of Mg are not over 200–300 g. Higher doses risk neuropathy from B6 or diarrhea from Mg. It is not expensive or especially difficult. It is credible that the genetic aberration resulting in autistic symptoms might involve a metabolic need for more than usual intake of these two nutrients. Therefore, a carefully monitored trial with moderate doses passes the SECS criterion and is acceptable.

### 2.3. Methyl B12

 Deficiency of methyl B12 (methylcobalamine) may occur in some people with ASD due to poor dietary intake, poor absorption, or metabolic dysregulation. Methyl B12 is a vital cofactor for the regeneration of methionine from homocysteine by providing methyl groups for the transmethylation and transsulfuration metabolic pathways. Reduced synthesis of the products of the transsulfuration pathway, including cysteine and GSH, may consequently lead to decreased antioxidant capacity. Glutathione dysregulation may be of particular significance, as GSH is a key antioxidant responsible for minimizing macromolecular damage produced by oxidative stress. Improvements may be noted in social relatedness, language, and behavior problems. Methyl B12 is often administered at high dose subcutaneous injections every 2 to 3 days. There are no studies of oral or nasal methyl B12, which are thought to be less effective because they do not keep consistently high levels. 

 James et al. [49] showed that many children with ASD exhibit low levels of GSH and a decreased GSH/GSSG redox ratio. Further, in a small, open-label trial, one-month administration of methyl B12 resulted in a significant increase in plasma GSH concentrations, although behavioral assessments were not done in this study [50]. Thirty subjects completed a 12-week, double-blind study of subcutaneously injected methyl B12 at a dose of 64.5 mcg/kg given every 3 days and 22 subjects completed the 6-month extension study [51]. No statistically significant mean differences in behavior tests or in glutathione status were identified between active and placebo groups. However, nine (30%) subjects demonstrated clinically significant improvement on the Clinical Global Impression-Severity Scale and at least two additional behavioral measures. More notably, these responders exhibited significantly increased concentrations of GSH and GSH/GSSG. The supplement was well tolerated. 

 The study mentioned above showing the response to methyl B12 of a subgroup of children with autism is the only published RCT but a new RCT from the same group is being presented at national meetings and will be completed in early 2013. Additional research is needed to delineate a subgroup of responders and ascertain a biomarker of response to methyl B12. Subcutaneous injectable methyl B12 does not meet SECS criteria because it is invasive (not easy) and based on only one published, controlled trial. But it does appear safe from this and other reports. While initial studies are promising for a subgroup of children with ASD and supplementation is well tolerated, additional study is needed to determine whether this is a recommended Tx for ASD.

### 2.4. Multivitamin/Mineral Supplements

 Although multivitamin and mineral levels generally are not found to be abnormal in children with autism, biomarkers of nutritional status have been reported and are found to be associated with autism severity [52]. There are only two clinical trials of vitamin/mineral supplements for children with autism, both from the same group. The first randomized 20 children aged 3–8 yr with ASD to a 31-ingredient vitamin/mineral formula (*n* = 11) versus placebo (*n* = 9) for 3 months [53]. The doses ranged from below the RDI/RDA (Recommended Daily Intake/Recommended Dietary Allowance) to 10 times as much. B6 was 30 mg as pyridoxal-5-phosphate, magnesium was 200 mg, and zinc 15 mg. The micronutrient supplement yielded significantly better sleep and gastrointestinal symptoms than placebo.

 A RCT of oral vitamin/mineral supplement for 3 months with 141 children and adults with ASD showed improved nutritional and metabolic status of children with autism, including improvements in methylation, glutathione, oxidative stress, sulfation, ATP, NADH, and NADPH [54]. The supplement group had significantly greater improvements than did the placebo group on the Parental Global Impression-R Average Change (*P* = 0.008), Hyperactivity (*P* = 0.003), and Tantruming (*P* = 0.009).

 A clinic study reported on 44 patients with ASD treated with the vitamin-mineral mix at parent preference (6 were actually treated with prenatal vitamins, available on Medicaid prescription, to save the family money) [55]. The author had a large practice with ASD in which he tracked progress with periodic ratings. He treated most patients conventionally, but some parents did not want to use conventional medication. Upon accumulating 44 cases with 3–6 month Tx, he matched them with 44 conventionally treated patients (taking antipsychotics, SSRIs, and stimulants) on sex, age, initial severity, and duration of Tx. The micronutrient mix yielded significantly better results on the Aberrant Behavior Checklist (ABC, including Irritability Subscale), Clinical Global Impression (CGI), and self-injury; but medication was better on no measure. There were significantly less side effects with micronutrients. This study is not conclusive because it was not randomized, and parent preference might be associated with other prognostic factors. However, initial severity was matched.

 In summary, there is limited evidence for the efficacy of vitamin and mineral supplements for ASD although there is widespread usage. The promising results from the open label and 2RCT warrant larger, placebo-controlled RCTs with pre- and postmeasures of vitamin, mineral, and metabolic status. Meanwhile, multiple-vitamin and micronutrient supplementation passes the SECS criterion as long as no ingredient is above the upper tolerable limit. It is recommended for those with a restricted or idiosyncratic diet and those with poor appetite, and is acceptable for all others.

### 2.5. Folic Acid

Folic acid has been considered because a polymorphism in the gene for methylenetetrahydrofolate reductase (MTHFR C677T) doubles the risk of autism [56]. Expected improvements are on core autism symptoms such as communication. An open trial of folinic acid and B12 in children with ASD and antibodies to the cerebral folate receptor showed significant improvement in receptive and expressive language [49]. It is not clear whether folate or folinate would be the preferred supplement or whether adjunctive B12 is needed. Limitations to be addressed by future research include small sample and lack of randomization and blinding. Given the relative safety, this appears to pass the SECS criterion despite the uncertainly and lack of placebo-controlled evidence, but if tried it should be monitored closely for possible unexpected side effects.

### 2.6. Omega-3 Fatty Acids

 Omega-3 long-chain fatty acid supplementation is reasonable to consider because omega-3 fatty acids are essential to brain development [57], being part of optimal neuronal membranes and being substrate for production of eicosanoids (e.g., prostaglandins) necessary for cell communication and immune regulation, and low levels have been reported in children with ASD[58–60]. The two omega-3 acids of interest are eicosapentaenoic acid (EPA) and docosahexaenoic acid (DHA). Based on data from other disorders, they would be expected to improve mood, attention, and activity level as well as possibly autism symptoms.

 There have been 4 open trials [59, 61, 62] and 2 double-blind, placebo-controlled randomized pilot trials [63, 64]. Meiri et al. [61] openly gave 10 children aged 4–7 years 1 g daily of omega-3 for 12 weeks and reported that 8 of the 10 improved by 1/3 on the Autism Tx Evaluation Checklist, with no side effects. Politi et al. [62] openly gave 19 adults with ASD 0.93 g/day of EPA+DHA and 5 mg/day of vitamin E and observed 6 more weeks. They reported no effect at the end of Tx but a nonsignificant delayed benefit. Meguid et al. [59] openly gave 30 children aged 3–11 Efalex (240 mg DHA, 52 mg EPA, 48 mg gamma-linolenic acid, and 20 mg arachidonic acid daily) for 30 months. The Child Autism Rating Scale (CARS) score was reduced by 17% (*d* = 2, *P* = 0.0001). In 10 nonresponders, the CARS score correlated with alpha-linolenic acid, negatively with DHA, suggesting a desaturase enzyme deficiency. Johnson et al. [63] randomized 25 children (mean age 3.5 yr) with ASD to open DHA 400 mg/day or healthy low-sugar diet for 3 months. There was little to no effect in either group. Amminger et al. [64] randomized 13 children aged 5–17 yr to 840 mg eicosapentaenoic acid (EPS) and 700 mg DHA per day (*n* = 7) or placebo (*n* = 6) for 6 weeks. There were no significant differences between groups on the ABC, possibly due to small sample and insufficient power, but omega-3 appeared nominally superior to placebo for stereotypy (*d* = 0.72), hyperactivity (*d* = 0.71), and inappropriate speech (*d* = 0.39). Bent et al. [65] randomized 27 three–eight year-olds with ASD to 700 mg EPA and 460 mg DHA per day (*n* = 13) versus placebo (*n* = 12) for 12 weeks. There was no significant difference between groups on the ABC, but with the small sample, power may have been insufficient. Hyperactivity decreased by 2.7 points with omega-3 versus 0.3 points with placebo (*d* = 0.38). There was no difference in side effects. 

 With only 2 small placebo-controlled RCTs totaling 38 children, all 3 without statistically significant effects (possibly a power issue), the evidence is rather thin for omega-3 supplementation in ASD. Limitations to be addressed by future studies include sample size, necessary duration of Tx, dose, and ratio of EPA to DHA (one of the failed pilot studies used only DHA). Nevertheless, it is safe, easy, cheap, and sensible in light of the known nutritional need for omega-3 fatty acids and their benefit for cardiovascular health, ADHD, and mood disorders. Thus, it passes the SECS criterion and is acceptable for ASD while awaiting definitive research.

### 2.7. Probiotics and GI Medication

 There is increasing evidence for a gut-brain connection associated with at least some cases of ASD [66]. This suggests benefit from a comprehensive digestive enzyme and probiotics with meals to aid digestion of all exorphin peptides and disaccharides, especially for those with gastrointestinal (GI) disturbance. Probiotics are microorganisms thought to improve digestive health. Some would suggest that these agents may also help to remove toxins and help with immune function.

 A double-blind placebo-controlled trial using crossover design over 6 months for 43 children with ASD, aged 3–8 years, did not show any clinically significant improvement of ASD symptoms with enzyme use [67]. However, possible effects on improvement in food variety suggest further detailed investigation. Curemark (http://www.curemark.com/) notes that it has reached its targeted enrollment for their CM-AT Phase III of a total 170 children with autism at 18 sites. CM-AT targets enzyme deficiencies that affect the availability of amino acids in children with autism. Curemark's autism therapy has received Fast Track review status from the FDA. There are no trials of probiotics for ASD reported. Testimonial evidence is that coordinated use of probiotics significantly increases clinical success in normalizing gut flora in people with ASD. Enzyme treatment and probiotics are proposed to improve self-stimulation and stereotypies, aggression, GI symptoms, socialization, and hyperactivity. Research limitations and future directions include the lack of large double-blind placebo-controlled trials with long-term follow-up. The published results from the Curemark study will likely give much more information.

 While there is no published evidence that probiotics or digestive enzymes are effective in treating ASD, their use for treating GI symptoms and their safety profile suggest that they might be considered in treating individuals with ASD and GI symptoms.

### 2.8. Iron Supplementation

 Low serum ferritin and low iron intake are reported in some children with ASD [68]. Low levels are associated with psychomotor retardation, poor sleep, and neurological and behavior problems, which might be logical targets of iron supplementation. Recent investigations suggest that antipsychotic Tx, commonly used for irritability of autism (with FDA-approved indication), is associated with reduced body iron stores (Chadi Calarge, personal communication). An 8 week open trial of 6 mg oral iron per day with 43 children with ASD aged 2–10 with 33 completers found that 69% of the preschool and 35% of the school-aged children had low serum ferritin and dietary iron intake and 79% had restless sleep [69]. With supplementation, mean ferritin increased significantly (16 microg/L to 29 microg/L), as did mean corpuscular volume and hemoglobin, suggesting that low ferritin in this patient group resulted from insufficient iron intake. There were significant improvements in Restless Sleep score but not Sleep Delay or CGI scores. With only an open trial with 33 completers, a larger, double-blind, placebo-controlled RCT with follow-up, multiple assessment domains, and multiple measures of iron deficiency is needed to know the full extent of iron deficiency and supplementation effects in children with ASD. Iron supplementation is safe and sensible for those ASD children *with low serum ferritin*, easy and cheap, and is therefore recommended for this subgroup. It also would be reasonable to screen children with ASD for iron insufficiency. At the current state of knowledge, it should not be used above the RDA amount without evidence of low iron.

### 2.9. Chelation

 Chelation is a process for removing heavy metals from the blood and is used in treating ASD based on the unproven theory that ASD is caused by heavy metal toxicity. The accumulation of heavy metals, particularly mercury, is theoretically due to either the body's inability to clear the heavy metals or to increased exposure or both. Detoxification involves courses of oral DMSA (2, 3 dimercaptosuccinic acid) with periodic elemental analysis of urine from subjects and controls. To be successful, detoxification Tx requires two prerequisite Txs that must be successful—clearing the gut of harmful dysbiotic flora, and bolstering metabolism with essential nutrients so that the individual can tolerate detoxification.

 Two related studies have been published [70, 71] involving 65 children with ASD who received one round of DMSA (3 days) and based on those who had high urinary excretion of toxic metals, 49 were randomly assigned to a double-blind design to receive either 6 additional rounds of DMSA or placebo. DMSA was reportedly well tolerated and resulted in high excretion of heavy metals, normalization of red blood cell glutathione, and possibly improved ASD symptoms. However, excretion of heavy metals and improvement only occurred after one round of DMSA with the additional six rounds being no better than placebo. Subjects demonstrated improvements in language, cognition, and sociability. Clearly, further studies, including randomized, placebo-controlled trials, are indicated to confirm these results.

 Regarding anecdotal evidence, of all the drug, diet, and nutritional therapies listed on the ARI Survey, detoxification is reported to help the highest percentage of individuals with ASD (71%) and it “worsened” only 3%, the second lowest percentage. Those who favor chelation are clinicians who are knowledgeable and experienced with it.

 However, chelation is controversial and the Institute of Medicine (IOM) recently warned of unspecified “risks.” Renal and hepatic toxicity is possible with oral agents. Most common side effects are diarrhea and fatigue. Less common side effects include abnormal complete blood count (CBC), Liver Function Tests (TFTs), mineral abnormalities, seizures, sulphur smell, regression, GI symptoms and rash. Therefore, we only recommend chelation for ASD if heavy metal toxicity is confirmed.

### 2.10. L-Carnosine

 L-Carnosine has been considered because it can be neuroprotective or improve function of frontal lobes. In an 8-week double-blind RCT with 31 children aged 3–12 with ASD, l-carnosine (800 mg/day) but not placebo showed statistically significant improvements on the Gilliam Autism Rating Scale (total score and the Behavior, Socialization, and Communication subscales) and the Receptive One-Word Picture Vocabulary test (all *P* < .05) [72]. Hyperactivity and excitability were the main side effects. This is the only study of l-carnosine for the Tx of autism. Additional studies replicating these findings with large double-blind placebo-controlled studies are necessary to recommend this Tx. Considering the side effects and equivocal evidence of efficacy, l-carnosine is borderline on the SECS criterion. It would be towards the bottom of a preference list and if tried, it should be monitored closely.

### 2.11. Ascorbic Acid

 The rationale for ascorbic acid (vitamin C) supplementation in large doses is that it blocks binding to DA receptors and possibly corrects redox balance, leading to correction of metabolic stress that may contribute to autism symptoms. In a 20 wk double-blind crossover following a 10-week single-blind ascorbate run-in, 18 residential patients aged 6–19 years were randomized to ascorbate-placebo or placebo-ascorbate order [73]. The dose was 90 mg/kg (8 g/day for 70 kg person). Both double-blind placebo conditions were actually ascorbate withdrawal states. There was a significant difference between ascorbic acid and placebo on the Ritvo-Freeman Real-Life Rating Scale, mainly consisting of improvement in stereotypy. The authors distinguished 3 subgroups: strong responders, modest responders, and nonresponders.

 Because ascorbic acid doses this large could interfere with B12 absorption, there is some risk, which could be ameliorated by additional B12, but the amount needed is not established. Due to this safety issue (as well as efficacy) ascorbic acid in these megadoses requires further study, does not currently pass the SECS criterion, and is not recommended.

### 2.12. Cyproheptadine

 High levels of 5-HT have been reported in ASD so Tx with cyproheptadine, a 5-HT2 antagonist, has been proposed. A double-blind, placebo-controlled study with 40 children using haloperidol in each Tx arm found that cyproheptadine was well tolerated and showed greater benefit on two scales (ABC and CARS) than did haloperidol plus placebo [74]. However, cyproheptadine has some risk and recommended use awaits replication and a larger number of subjects assessing core symptoms of autism, disruptive behaviors, and physiological symptoms. 

### 2.13. Immune Therapies

 Evidence is accumulating that ASD subgroups have immune deficiencies and autoimmunity [75]. Various approaches have been tried to boost immune function or block autoimmunity. One of the most obvious has been immune globulin (IVIG) but the results have been weak. In one open-label study IVIG Tx improved eye contact, speech, behavior, echolalia, and other autistic features [76]. Others have claimed that IVIG Tx led to improvements in GI signs and symptoms, as well as behavior.

 Currently there are six published open-label trials of IVIG Tx with ASD. Other than Gupta's study [76] finding promising results for IVIG Tx, subsequent studies have reported questionable benefits and mixed results for language and behavior. It is unclear if an underlying immunological dysfunction is present in all individuals with ASD or if Tx should target the inflammatory changes and this CAT has some risk. Therefore, IVIG therapy is not recommended for the Tx of ASD. Other immune boosting therapies may be of benefit but have not been adequately studied.

## 3. Noningestible CATs for ASD

### 3.1. Massage Therapy

This CAT involves the manipulation of superficial layers of muscle and connective tissue to enhance bodily functioning, relaxation, and well-being. It has been suggested for ASD to increase connectivity to others and reduce overarousal. Five RCTs (all single blind) have examined massage therapy [77–81] and there is one systematic review [82]. Collectively these studies involved 204 one-to-fifteen year olds, receiving massage therapy 10–60 minutes, 1-7X/week over 3–32 weeks. Reported results include significantly improved total ASD symptoms, social relatedness, sleep, language, social communication, and receptive language and significantly reduced ADHD symptoms, repetitive behaviors, sensory issues, disruptive behavior, and anxiety. Therefore, based on this research, similar clinical outcomes are predicted for massage therapy for youth with ASD. Research limitations and future directions include the lack of large double-blind, sham RCTs with long-term follow-up. As massage therapy appears safe, easy, cheap and sensible, if parents are trained to administer it is likely to improve the parent-child relationship, and is therefore recommended.

### 3.2. Acupuncture

 Based in Traditional Chinese Medicine, acupuncture involves the systematic insertion and manipulation of thin needles into the body, via 400 acupoints, to improve health of body/mind by unblocking the flow of qi (“energy”). For ASD, there are three RCTs (1 DB sham controlled) published in English using scalp [83], tongue [84], and electro acupuncture [85]. These RCTs examined 125 three-to-thirteen year olds, via evidence-based assessment of ASD, using acupuncture for 15 seconds–30 minutes, 2–5X/week, over 4–36 weeks, while monitoring possible adverse effects. All these types of acupuncture were reported to be tolerated by >80%, with few or mild adverse-effects. Reported significant results and, therefore, expected clinical outcomes for this Tx include improvement in attention, receptive language, self-care, language, overall functioning, and communication. Although published after completion of our literature search, it is important to note a recent review of acupuncture for ASD because it includes a further 9 RCTs (mean *N* = 45 [range 30–70], 1 single blind) published in Chinese only [86]. Although we could not probably examine this review as it was in Chinese, its English abstract review reported significant “behavioral and/or developmental improvements” (p.1). Therefore, based on all 12 RCTs, acupuncture has overall tolerability but highly variable Tx presentations.

 Research limitations and future directions include the lack of double-blind, sham-controlled RCTs, long-term follow-up, standard Tx protocols, use of standard Tx outcome measures, and monitoring of possibly confounding concomitant Txs. As acupuncture appears safe and seems sensible (from a Traditional Chinese Medicine perspective) and easy, it is acceptable for ASD if not too expensive.

### 3.3. Exercise

 Exercise programs have been found to be beneficial for a variety of psychiatric and developmental disorders [87]. In children with ASD, exercise may reduce hyperactive and repetitive behavior through the release of certain neurotransmitters, such acetylcholine, or beta-endorphins [88]. Antecedent aerobic exercise (the individual participates in a short period of vigorous aerobic exercise prior to a learning task or observational period) has been the most widely studied exercise intervention for children and adults with ASD. Eight within-subject studies (*N* = 36) compared the benefit of antecedent aerobic exercise (e.g., jogging, ranging from 6 to 20 minutes) to nonaerobic exercises antecedents (e.g., academic tasks, walking) [89–96]. and one review has been published [97]. Seven out of eight studies found that antecedent aerobic exercise decreased self-stimulatory behavior, and two out of three studies found increased academic performance following aerobic exercise. Research limitations and future directions include the lack of double-blind, sham-controlled RCTs, long-term follow-up, standard Tx protocols, use of standard Tx outcome measures, and monitoring of adverse and concomitant Txs. Antecedent exercise seems sensible, cheap, safe, and easy and is therefore acceptable, before academics or play, if feasible for the child and setting, particularly those with significant repetitive behavior. Several other studies have investigated exercise programs for children with ASD (e.g., swimming lessons, treadmill walking); while many reported increases in sports skills and/or physical fitness, the impact of these types of programs on ASD symptoms is not well evaluated. 

### 3.4. Music Therapy

 Music therapy involves structured and unstructured individual and group sessions with and without a leader involving playing and/or listening to music. It has been used to Tx ASD because of its potential for assisting communication, joint attention, expression, engagement, and relationships with the environment [98]. Most research on music therapy involves case studies with only two randomized single-blind, repeated measures, within-subject comparison designs [99–101]. These studies had a total of 20, 3-to-9 year-olds with ASD, with varied Tx presentations, given 1–20X/week for 1–12 weeks for 30 minutes. Significant results and potential clinical outcomes include improvement in imitating signs and words, longer and more eye contact and turn-taking, joint attention, nonverbal communication, longer and more “joy,” emotional synchronicity, initiating engagement and compliant behavior. Research on music therapy for ASD lacks evidence-based assessment of ASD, large samples, RCTs, standardized protocols, double-blind, sham, use of standard Tx outcome measures, follow-up, monitoring of adverse-effects, or concomitant Txs. However, it appears safe, seems sensible, easy and cheap and is therefore acceptable.

### 3.5. Animal-Assisted Therapy (AAT)

 AAT involves structured and supervised therapeutic interaction with animals, which are seen as transitional objects for initial bonding for individuals with ASD before generalizing this attachment to people. Although there are many case studies of AAT, only four studies have recruited multiple subjects, with the most recent being the only RCT [102–105]. Collectively, these studies included 69, four to thirteen year olds, given AAT 15–60 minutes, 1-3X/week over 12–16 weeks. Reported results included significant improvement in playful mood, focus, awareness of social environment, use of language, social interaction, and motivation to interact with the environment, all of which are hoped to occur with clinical application of AAT for youth with ASD. AAT research lacks large RCT, double-blind, evidenced-based assessments of ASD standard Tx outcomes, follow-up, and monitoring of adverse effects and concomitant Txs. AAT appears safe (if done under trained supervision) sensible and possibly easy but it may be expensive, so it is therefore potentially acceptable as a CAT for ASD.

### 3.6. Neurofeedback (NF)

 NF “trains” the brain to improve self-regulation of itself by providing it with real-time video/audio information about its EEG activity. It has been suggested for the Tx of ASD because QEEG studies indicate over- and underconnectivity [106] and a wide variety of significant EEG differences associated with ASD have been reported [107]. One review [108] and three RCTs have been published on NF for ASD. These RCTs involved 47 seven to seventeen year-olds with ASD ([109, 110]), all used Evidence-Based Assessments and provided NF for 21–30 minutes session, 2-3/week for 10–20 weeks. Both Pineda studies [109] used a sham-NF control, with the first study (*N* = 8) a single blind and the second study a double-blind design. NF was focused on increasing mu suppression, an EEG correlate of mirror-neuron activity associated with imitation abilities, thought to be limited in ASD. In the first study, compared to controls, mu suppression (EEG correlate of self-imitation) was significantly increased, and Tx increased sustained attention and improved scores on the sensory/cognitive awareness subscale of the parent-rated Autism Treatment Evaluation Checklist (ATEC). Similarly, the second larger study also reported significant improvements in sustained attention and parent-rated ATEC speech/language communication, sociability, health/physical behavior subscales and overall score (but not, curiously enough, for sensory-cognitive subscale), and increased mu suppression. Neither study showed the expected significant behavioral improvements in imitation by the Tx group.

 Kouijer et al. [110] used a waitlist control (*N* = 20 in each condition) and reported significant (compared to control) reductions in excessive theta power (reflecting change of activity in the anterior cingulate cortex that thought to be involved in ASD social and executive problems); significant improvements in parent-rated reciprocal social interaction and communication skills, and significant improvement in neuropsychological set shifting skills. Six-month maintenance of Tx gains and some improvements was demonstrated for the Tx but not control group. 

 Based on these 3 studies, the following clinical improvements are expected with sustained attention and set-shifting skills; parent-rated speech/language communication, sociability, health/physical behavior, reciprocal social interaction, communication skills, and, possibly, sensory/cognitive awareness. Research limitations and future directions include the lack of a large double-blind RCT sham study with follow-up, use of standard Tx outcome measures, monitoring of adverse effects, and concomitant Txs. Even though NF appears safe (although not empirically examined) and seems sensible, it is not easy or cheap and is therefore not recommended at this time. 

 Finally, the following CATs (with cited reviews) are not recommended because they failed to show positive effects across several RCTs: Auditory Integration Therapy [111], Facilitated Communication [112], Gluten/Casein-Free Diet [36], Hyperbaric Oxygen Therapy [113, 114], and Secretin [115]. Furthermore, we do not recommend packing therapy or faradic skin shock due to ethical/safety issues.

## 4. Summary

Nineteen CATs were reviewed, including 13 ingestible and 6 noningestible CATs. Research on these CATs is extremely varied, ranging from case studies to double-blind, sham-controlled RCTs, with and without significant results. Their safety, easy of use, sensibility, and expense (SECS) also vary considerably. Currently, we would only recommend two ingestible and one noningestible CAT, melatonin and RDA/RDI multivitamin/mineral (for those with a limited diet and/or poor appetite), and massage therapy, respectively. However, the following CATs are considered acceptable and worth considering for a short, monitored trial, if conventional Txs for ASD and the two recommended CATs have been given a reliable trial and found ineffective. For ingestible CATs: B6 and magnesium, multivitamin/mineral (even without a restricted/idiosyncratic diet and/or poor appetite, as long as no ingredient is above tolerable limit), folic acid, omega-3, L-Carnosine, probiotics and GI medication (only for ASD patients with GI symptoms), iron supplementation (only for those with low serum ferritin), and chelation (only for those with confirmed heavy metal toxicity). For noningestible CATs: Acupuncture, exercise, music therapy, and animal-assisted therapy.

 Although published after our literature search, we feel it is important to mention N-Acetylcysteine (NAC) as an ingestible CAT that has great potential. NAC is a glutamatergic modulator and antioxidant and was recently examined in a 12-week, double-blind, randomized, placebo-controlled study in children with autistic disorder [116]. Thirty-three, 3–10 year-olds were randomized and NAC was initiated at 900 mg daily for 4 weeks, then 900 mg twice daily for 4 weeks and 900 mg three times daily for 4 weeks. Oral NAC was well tolerated with limited side effects and compared with placebo, resulted in significant improvements on the ABC irritability subscale (*F* = 6.80; *P* < .001; *d* = .96).

## 5. Future Research

This review of ingestible and noningestible CATs for ASD indicates to us that this promising field is in need of more double-blind, placebo/sham controlled RCT's with long-term follow-up and reliably diagnosed samples of participants with ASD, using standard Tx outcome measures, while continually monitoring potential adverse effects and changes in concomitant Txs. We also encourage definitive trials and replications of the more promising CATs that may have some advantage (benefit-risk ratio) over standard Txs if proven effective. For well-considered hypotheses for which there are no pilot data, we would suggest controlled clinical trials when easy and cheap and open pilot trials when controlled trials would be expensive or difficult. Finally, to fully realize the potential of these CATs, studies are required to compare them to established Txs (i.e., behavior modification and medicine) to examine incremental effects to designate CATs as complementary Txs and similar effects to designate CATs as alternative Txs.

## 6. Clinical Recommendations

 Although current research on CATs for ASD is limited, the Tx needs of individuals cannot always wait for science to improve. So for those who do not respond completely to evidence-based Txs, practitioners are often expected to advise patients about CATs. As many families may also experiment with CATs by themselves, especially if they sense a practitioner's reluctance to consider CATs, we believe it is clearly better for individual trials to be guided professionally. In that respect, we offer practitioners the following 10 clinical recommendations.

(1) As many CATs target specific causes, they should be *considered* (not necessarily implemented) early during the diagnostic evaluation. Therefore, a detailed medical, psychological, developmental, family, Tx and dietary history, physical exam and, as indicated, a complete blood count, electrolyte/mineral screen, and, in areas with high rates of subclinical lead poisoning, serum lead assessment are needed. Once causes amenable to specific Txs are ruled out, standard generic Txs (behavior modification and medication) may be more confidently implemented.
(2) Clinicians and parents/patients need to request data/evidence as many commercially advocated Txs claim to have scientific proof, but can provide only anecdote, case history, or testimonial information.
(3) In Tx outcome research, comparisons to established Txs are not convincing unless assignment is random to control for selection effects and associated expectations due to subjects and parents choosing the preferred Tx, nonrandom subject experiences (i.e., subject history), regression to the mean, maturation, practice with assessments, or an interaction of any of these factors.
(4) Failure to find a significant difference from an established Tx does not make the Tx equal or effective because a small sample can easily fail to find a significant difference when one really exists.
(5) For some individuals, a CAT may work better than established Tx even though it is not demonstrated by group averages.
(6) The decision to try a CAT in an individual case depends partly on data, SECS criterion, and accessibility and response to conventional Txs.
(7) Diverting patient resources (money, time, and effort) to a Tx that does not work is a risk that needs to be considered for the patient and other family members.
(8) Change one thing at a time and monitor results and side effects with specific ASD rating scales or a simple graph (e.g., no. of weeks of Tx along the *x*-axis and effect of CAT along the *y*-axis, 0 [not helpful]-5 [moderately helpful]-10 [very helpful]). If appreciable benefit is not observed in the expected time, move on to another Tx.
(9) Patients and their families should be encouraged to discuss with their prescribing doctor all ingestible CATs, even “natural” herbs, to identify any possible interactions with currently prescribed medications or other ingestible CATs.
(10) Patients (as developmentally appropriate) and their families should be provided with biopsychosocial information or psychoeducation about ASDs and their Tx. As “knowledge is power,” psychoeducation, which is actually another form of complementary Tx, can give “power” to help people to improve cognitive and emotional control over a condition; alter cognitive schema to correct past misinformation and prevent future errors; access and collaboratively utilize cost-effective mental health, educational, and community services. Psychoeducation resources can be accessed from:
  - *National Institute of Health (NIH) National Center for Complementary and Alternative Medicine* http://nccam.nih.gov/;
  - *Autism Speaks* http://www.autismspeaks.org/;
  - *Autism Science Foundation* http://www.autismsciencefoundation.org/;
  - *Interactive Autism Network (IAN)* http://www.IANresearch.org/;
  - *Research Autism* http://www.researchautism.net/pages/welcome/home.ikml;

## Figures and Tables

**Table 1:** Commonly used complementary and alternative treatments (CATs) for autism spectrum disorders (ASD).

Name	Rationale/mechanism	Data type	Significant improvements and clinical indications	Potential adverse effects	SECS	Recommendation*
**Ingestible**						
Melatonin	↑ sleep	5 DB PBO RCT x-overs	Sleep duration and, overall sleep, sleep onset latency,	Minimal to none	S, E, C, S	R for sleep
B6/Mg	Correct deficiency	6 DB PBO RCT x-overs	Social interaction, communication, and repetitive behavior (in some but not all RCTs)	>200–300 g neuropathy diarrhea	S for <200 mg, E, C, S	A
B12	Correct deficiency	1 DB RCT	Some functioning and behavior	None reported	S, S but not C, E	NR b/c not E and sparse data
Multivitamin/mineral	Nutritional status associated with ASD sxs	2 DB PBO RCTs, 1 open label	Sleep, gastrointestinal, functioning, hyperactivity, tantrums, and self-injury	None reported	S (if RDA/RDI) E, C, S	R for RDA/RDI if poor diet;
Folic acid	Genetic abnormality	1 open label	Receptive and expressive language	None reported	S, E, C, S	A
Omega-3	Correct deficiency	2 DB RCTs 4 open trials	Stereotypy, hyperactivity, and inappropriate speech	None reported	S, E, C, S	A
Probiotics and GI medication	Remove toxins ↑ immune function	1 DB PBO RCT	None	None reported	Safe, E	A when GI problems
Iron supp.	Correct deficiency	Open trial	Restless sleep	None reported	S (only if low Fe) E, C, S	A if iron deficiency confirmed
Chelation	↓ toxic heavy metals	1 DB PBO RCTs	Language, cognition, and sociability	Renal/hepatic toxicity, fatigue, diarrhea	Sometimes not safe; not cheap	A if heavy metal toxicity confirmed
L-Carnosine	Neuroprotective ↑ frontal lobe functioning	1 DB PBO RCT x-over	ASD sxs and receptive/expressive language	Hyperactivity and excitability	E, Sensible	Possibly A
Ascorbic acid (vit C) 90 mg/kg	Corrects redox balance	1 DB PBO RCT x-over	Repetitive behavior	Can interfere with B12 absorption	E, C, S	NR in these doses
Cyproheptadine	High 5-HT levels	1 DB PBO RCT	ASD sxs	None reported	Some risk	NR b/c some risk and sparse data
Immune therapies	Immune deficiencies	6 open label	Some ASD sxs	None reported	Some risk	NR b/c some risk

**Noningestible**						
Massage	↑ attachment and ↓ overarousal	5 RCTs all SB	ASD sxs, sleep, social relatedness, social communication, speech rate, receptive language and ADHD sxs, sensory issues repetitive beh., anxiety, disruptive beh.	Not examined	S, E, C, S	R
Acupuncture	Unblocking flow of energy (“qi”)	3 RCTs** (1 DB, 2 SB)	Attention, receptive language, overall functioning, self-care, and communication	Few or mild possible infection, bleeding	S, E, S	A
Exercise	↓ hyperactive and repetitive behavior	8 within-sub.	Self-stimming and academic scores	Not examined	S, E, C, S	A
Music therapy	↑ verbal and nonverbal communication and engagement	2 randomized counterbalan. designs	Imitating signs and words, eye-contact turn-taking, joint attention, and nonverbal communication	Not examined	S, E, S	A
Animal-assisted therapy	↑ attachment	1 RCT 3 multiple case studies	Playful mood, focus, language use and social-awareness, interaction and motivation	Not examined; possible bites, scratches, accidents	S, E, S	A b/c may not be C
Neurofeedback	EEG changes	3 RCTs 1 SB, 1 DB	Attention, set-shifting, speech/language, sociability, health/physical behavior, reciprocal social interaction, communication, sensory cognitive awareness ↑ EEG mu suppression and ↓ EEG theta	Not examined	S, S	NR b/cnot C

Note. SECS: safe, easy, cheap, and sensible; *recommendations for an individual and monitored patient trial following procedure reported in study: R: recommended, A: acceptable, NR: not recommended; DB: double blind; PBO: placebo controlled; RCT: randomized clinical trial; X-over: crossover; sxs: symptoms; SB: single blind; **3 English-language articles that we could review thoroughly but 9 RCTs in Chinese only also exist.

**Table 2:** Randomized controlled trials of ingestible complementary and alternative treatments for autism spectrum disorders (ASD).

Tx	Author, year	*N*, age, % male	ASD type and DSM Dx	Control	Tx dose*, duration	Results
Melatonin	McArthur et al. 1998 [117]	9, 4–17 0%	Rett/N (clinical dx)	PBO	2.5–7.5 mg x-over; 4 wks each	Sleep latency^2,5^ *P* < 0.05 Night waking^2,5^: ns Sleep time^2,5^: ns
					
	Garstang et al. 2006 [118]	11, 5–15 63%	ASD/N (clinical dx)	PBO	5 mg x-over: 4 wks each	↓ Sleep latency^2*∗∗*^ ↓ night waking^2*∗∗*^ ↑ sleep time^2*∗∗*^
					
	Wasdell et al. 2008 [119]	51 (16 ASD) 2–18 62%	ASD/N (clinical dx)	PBO	5 mg x-over; 10 days each	Sleep latency^5^ *P* < 0.01 Night waking^5^: ns Sleep time^5^ *P* < 0.01 Overall sleep^3^ *P* < 0.01 Family stress^2^: *P* < 0.05
					
	Wirojanan et al. 2009 [40]	18 (12 ASD) 2–15 89%	ASD/N (ADOS; ADI-R)	PBO	3 mg x-over; 2 wks each	Sleep latency^2,5^ *P* = 0.02 Night waking^2,5^: ns Sleep time^2,5^ *P* = 0.0001
					
	Wright et al. 2010 [120]	20, 4–16 80%	ASD/N (ICD-10)	PBO	2–10 mg x-over; 3 months each	Sleep latency^2^ *P* < 0.05 Night waking^2^: ns Sleep time^2^: *P* = 0.002 Disruptive behavior^2^: ns Communication^2^: ns Social^2^: ns Anxiety^2^: ns

B6/Mg	Martineau et al. 1985 [47]	16, 3–14 63%	AD/Y	Mg only	B6: 30 mg/kg Mg: 10–15 mg/kg x-over; 2 wks each	Overall improved in both conditions^3*∗∗*^
					
	Martineau et al. 1985 [47]	21, 3–14 81%	AD/Y	PBO	B6: 30 mg/kg Mg: 10–15 mg/kg x-over; 2 wks each	Improved ASD^3*∗∗*^
					
	Martineau et al. 1985 [47]	35, 3–14 57%	AD/Y	PBO	Mg: 10–15 mg/kg x-over; 2 wks each	No change ASD^3*∗∗*^
					
	Martineau et al. 1985 [47]	37, 3–14 57%	AD/Y	PBO	B6: 30 mg/kg/ x-over; 2 wks each	No change ASD^3*∗∗*^
					
	Lelord et al. 1981 [45]	21, 3–16 unk	Autistic Beh. N	PBO	625–1125 mg B_6_ 400–500 mg Mg x-over; 2 wks each	Evaluated if response/nonresponse status from OL remain in DB *P* < 0.01
	Findling et al. 1997 [46]	12, 3–17 92%	AD/Y	PBO	30 mg/kg B_6_ 10 mg/kg Mg x-over; 4 wks each	ASD^3^: ns Repetitive behavior^3^: ns ADHD^1,2^: ns

B12	Bertoglio et al., 2010 [51]	30, 3–8, 93%	AD/Y	PBO	64.5 mg/kg injection, every 3rd day, 12 wks	ASD^3^: ns^*∗∗*^ Language^5^: ns**

Micronutrients	Adams and Holloway 2004 [53]	20, 3–8 90%	ASD/N (clinical dx)	PBO	3 mL/5 lbs 3 months	Communication^2^: ns Social^2^: ns Sleep^2^ *P* = 0.03 Gastrointestinal^2^ *P* = 0.03
					
	Adams et al. 2011 [52, 54, 66]	141, 5–60 91%	ASD/N (clinical dx)	PBO	Formulated by Yahoo Health	ASD^2^: ns Expressive language^2^: ns Recep language^2^: *P* = 0.03 Gastrointestinal^2^: ns Sleep^2^: ns Social^2^: ns Hyperactivity^2^: *P* = 0.003 Disruptive beh^2^: *P* = 0.009

Omega-3	Amminger et al., 2007 [64]	13, 5–17 100%	AD/Y	PBO	840 mg EPA, 700 mg DHA 6 wks	Irritability^3^: ns Hyperactive^3^: ns Repetitive beh^3^: ns
					
	Bent et al. 2011 [65]	27, 3–8 89%	ASD/Y	PBO	700 mg EPA, 460 mg DHA 12 wks	Hyperactivity^2^: ns ASD^2^: ns Language^5^: ns Repetitive behavior^2^: ns

Probiotics	Munasinghe et al. 2010 [67]	43, 3–8 84%	ASD/Y	PBO	Up to 9 caps x-over; 3 months each	ASD^2^: ns Language^2^: ns Gastrointestinal^2^: ns Sleep^2^: ns Food variety^2^: *P* = 0.02

L-Carnosine	Chez et al. 2002 [72]	31, 3–12 68%	ASD/Y	PBO	800 mg 8 wks	ASD^2^ *P* = 0.01*** Receptive vocab^5 ^ *P* = 0.01*** Expressive vocab^5^: ns***

Ascorbic Acid	Dolske et al. 1993 [73]	18, 6–19 72%	AD/Y	PBO	90 mg/kg x-over; 10 wks each	Repetitive beh^4^ *P* = 0.01 Language^4^: ns Social^4^: ns Sensory^4^: ns

Cyproheptadine	Akhondzadeh et al., 2004 [74]	40, 3–11 60%	AD/Y	Haloperidol + PBO	0.2 mg/kg Cypro + Haloperidol 0.5 mg/kg 8 wks	ASD^2^ *P* = 0.004

Gluten-Free/ Casein-Free Diet (GFCF)	Knivsberg et al. 2003 [121]	20, *M* = 7.4 unk	AD/N (clinical dx)	No Tx	GFCF diet 12 months	Social^2^ *P* < 0.003 Language^2^ *P* < 0.004 Repetitive beh^2^ *P* < 0.007
					
	Elder 2006 [122]/Seung et al. 2007 [123]	15, 2–16 80%	ASD/Y	Typical diet	GFCF diet x-over; 6 wks each food provided, blinded	ASD^3^: ns Language^4^: ns Social^4^: ns
	Whiteley et al. 2010 [124]	72, 4–10 89%	ASD/N (ICD-10)	No Tx	GFCF diet 12 months	Social: ns^2,4^, *P* < 0.0001^2^ Communic: ns^2^, *P* = 0.002^4^ Repetitive beh^2,4^: ns ADHD^2^: *P* < 0.05
					
	Johnson et al. 2011 [125]	22, 3–5 82%	ASD/Y	Healthy diet	GFCF diet 3 months	ADHD^2^: *P* < 0.05 Communication^4^: ns Social^4^: ns

Note: AD: Autistic Disorder, ASD: Autism Spectrum Disorder; ASP: Asperger's Disorder; unk: unknown; Tx: treatment; dx: diagnosis; DSM dx: Y: yes (children diagnosed according to DSM-III or DSM-IV symptoms of ASD) and N: no (children not specifically diagnosed by DSM criteria; ADOS: Autism Diagnostic Observation Schedule; ADI-R: Autism Diagnostic Interview Revised); PBO: placebo; wk: week; x-over: cross-over; WL: Wait List; ns: nonsignificant; *treatment dosages are per day, unless otherwise specified; OL: open label; beh: behavior; vocab: vocabulary; communic: communication; results superscripts indicate the method of assessment: ^1^teacher/staff, ^2^parent, ^3^therapist/clinician, ^4^observation, ^5^psychometric/standardized testing; **significance values not included in the text; ***P values only provided for pre-post comparison for active treatment, not compared to placebo. *M*: mean.

**Table 3:** Randomized controlled trials of noningestible complementary and alternative treatments for autism spectrum disorders (ASD).

Tx	Author, year	*N*, age, % male	ASD type and DSM Dx	Tx info	Tx dose, duration	Control	Results
Massage	Field et al. 1997 [77]	22, *M* = 4.5 54%	AD/Y	Given by staff at school	30 min/wk, 4 wks	Attention (games)	ASD^1^ *P* < 0.05 Repetitive behavior^4^ *P* < 0.01 Social^5^ *P* < 0.05
						
	Escalona et al. 2001 [78]	20, 3–6 54%	AD/Y	Parent massage at bedtime	15 min/day, 4 wks	Reading at bedtime	ADHD^1,2,4^ *P* < 0.05 Repetitive behavior^4^ *P* < 0.05 Social^4^ *P* < 0.05 Improved sleep: sig^*∗∗*^
						
	Silva et al. 2007 [79]	15, 3–6 87%	AD/Y	Therapist 2x/wk Parent: daily	15 min/day 5 months	WL	Sensory^2^ *P* = 0.01 ASD^2^: ns Social^2^ *P* = 0.04 Language^2^: ns
						
	Silva et al. 2009 [80]	46. 3–6 80%	AD/N (eligible for AD services)	Therapist 2x/wk Parent: daily	15 min/day 5 months	WL	Social/commication^1,2^ *P* < 0.01 ASD^2^ *P* < 0.01 Sensory^2^ *P* = 0.002
						
	Piravej et al. 2009 [81]	60, 3–10 82%	AD/Y	SI Tx + massage by therapist	2 hrs/week 8 wks	SI Tx only	ADHD^1,2^: ns Disruptive behavior: ns^1^, *P* = 0.03^2^ Sleep^2^: ns Anxiety^2^ *P* = 0.01

	Allam et al. 2008 [83]	20, 4–7 60%	AD/Y	Scalp acup + speech Tx	2x/wk speech 40 min/wk acup 9 months	Speech Tx only	ADHD^3^ *P* = 0.21 Receptive language *P* = 0.034^5^, ns^5^ Expressive langauge^5^: ns
							
Acupuncture	Wong and Sun 2010 [84]	50, 3–11 88%	AD/Y	Tongue acup	5x/wk 8 wks	Sham	Social^2,5^: ns Language^2,4,5^: ns Sensory^4^: ns Cognitive: ns^5^, sig^3*∗∗*^ Adaptive: sig^3*∗∗*^
							
	Wong et al. 2010 [85]	55; 3–18 85%	ASD/Y	Electro acup	3x/wk 4 wks	Sham	Social: ns^2,3^, *P* = 0.01^2^ Sensory^2^: ns Communication^2,3,5^: ns Receptive language^2,3^: *P* < 0.05 Repetitive behavior^2^: ns Cognitive^2^: ns Overall function^2^ *P* = 0.003

Animal assist	Bass et al., 2009 [105]	34, 4–10, 85%	ASD/Y	Equine Tx	1 hour/wk 12 wks	WL	Sensory^2^ *P* = 0.002 ASD^2^ *P* = 0.04

Music therapy	Buday, 1995 [99]	10, 4–9 80%	AD/unk	Structured	5 sessions	Rhythm therapy	Communication^4^ sig (in Tx session)
						
	Kim et al. 2008 [100]/Kim et al. 2009 [101]	20, 3–6 100%	AD/Y	Music therapy Improv music therapy	30 min/wk 12 wks	Play with toys	Social: ns^2,3^, *P* = 0.01^4^ Social^4^ *P* < 0.001 (in Tx session) Comply^4^: *P* < 0.01 (in Tx session)

Neurofeedback	Pineda et al. 2008 [109] Study 1	8, 7–17 100%	ASD/N (clinical dx)	↑ Mu suppression	90 min/wk 10 wks	Sham	ADHD^5^ *P* < 0.02 Sensory^2^ sig^*∗∗*^ Social^2^: ns^*∗∗*^ Communication^2^: ns^*∗∗*^
						
	Pineda et al. 2008 [109] Study 2	19, 7–17 84%	ASD/N (ADOS; ADI-R)	↑ Mu suppression	90 min/wk 10 wks	Sham	Improvement in ADHD^5^, social^2^, and communcation^2^; not stated if significant^*∗∗*^; sensory not improved
						
	Kouijzer et al. 2010 [110]	20, 8–12 85%	ASD/Y	Excess ↓ theta	40 txs, 6 months	No Tx	ASD: ns^1^, ns^2^, *P* < 0.01^2^ Communication: ns^1^, *P* = 0.02^2^

Note: AD: Autistic Disorder, ASD: Autism Spectrum Disorder; DSM Dx: Y: yes (children diagnosed according to DSM-III or DSM-IV symptoms of ASD) and N: no (children not specifically diagnosed by DSM criteria; diagnosis method listed in parentheses); Tx: treatment; dx: diagnosis; ADOS: Autism Diagnostic Observation Schedule; ADI-R: Autism Diagnostic Interview Revised; wk: week; SI: sensory integration; acup: acupuncture; WL: Wait List; ns: nonsignificant; results superscripts indicate the method of assessment: ^1^teacher/staff, ^2^parent, ^3^therapist/clinician, ^4^observation, ^5^psychometric/standardized testing; **significance values not included in the text. *M*: mean.

**Table 4:** Complementary and alternative treatments for autism spectrum disorders either without positive effects in randomized control trials (RCTs) or without sufficient evidence to evaluate (listed alphabetically).

Without positive effects in RCTs		

Auditory integration therapy [111] Facilitated communication [112] Gluten/Casein-Free Diet [36] Hyperbaric oxygen therapy [113, 114] Secretin [115]		

Without sufficient evidence to evaluate		

Allithiamine Antibiotics and antifungals Atkins diet Betaine Bethanechol Bolles Sensory Learning Method Calcium Colostrum Craniosacral therapy Cysteine Daily life therapy Dairy/milk-free diet Deep pressure therapy Dimethylglycine Doman-Delacato Patterning Fast ForWord Feingold diet Flexyx Neuropathy System Fluconazole	Gentle teaching Giant steps Glutathione Homeopathic treatments Integrated movement therapy Interactive metronome Irlen method/lenses Ketogenic diet L-Glutamine Lindamood-bell learning processes Miller method Movement/dance therapy Neural therapy Osteopathic manipulation Prayer* Psalms* Reduced L-glutathione Rhythmic entrainment Rolfing/structural integration Selenium	Sensory integration therapy Specific carbohydrate diet Spiritual practices* Sporanox St. John's Wort Transfer factor Urecholine Vagal nerve stimulation Vision therapy Vitamin A Vitamin E (alpha-tocopherol) Vivitrol Watsu Weighted blanket/vest Yeast-free diet Zinc

Note: (1) List of CATs from Medline/PsychINFO reviews and Interactive Autism Network (IAN) research online parent questionnaire of treatments utilized for child with ASD http://www.iancommunity.org/cs/ian_research_questions/treatment_list.

(2) Although included in the IAN study, the following were not included in our CAT review because they are in the realm of conventional treatments such as speech, special education, and cognitive and behavior therapy: Augmentative and Alternative Communication (AAC), Floortime (from Difference, Relationship-based approach), Discrete Trial Training, Language Preschool, Lifeskills and Education for Students with Autism and other Pervasive Behavioral Challenges (LEAP), Picture Exchange Communication System (PECS), Pivotal Response Training (PRT), Rapid Prompting Method, Relationship Development Intervention (RDI), Self-Injurious Behavior Inhibiting System (SINIS), Social Stories, Treatment and Education of Autistic and Communication-Handicapped Children (TEACCH), Toilet Training, and Visual Schedules.

(3) Some of CATs in the table have evidence of positive effects in other disorders, but not for ASD. This does not necessarily mean they are without merit, just that there is not enough current evidence of a positive effect for ASD.

**(4) Due to ethical/safety issues we do not recommend packing therapy or faradic skin shock.**

*Although we respect individual's religious views, prayer, psalms, and spiritual practices, which were identified by parents as utilized treatments in the IAN survey, these approaches do not currently have any scientific evidence of a positive effect for ASD symptoms.
